# Up-regulated matrix metalloproteinase activity in soil-transmitted helminth–tuberculosis co-infection is associated with increased lung pathology

**DOI:** 10.1183/13993003.01445-2024

**Published:** 2025-01-30

**Authors:** Maria-Cristina I. Loader, Sory Vasquez Alves, Robert H. Gilman, Jorge Coronel, Carmen Taquiri, Neusa Vasquez Alves, Fabiola Díaz-Soria, Salomón Durand, Sean T. Kelleher, Teresa Jacob, William H. Elson, Daniela E. Kirwan, Jon S. Friedland

**Affiliations:** 1 Institute for Infection and Immunity, St George's University of London, London, UK; 2 Department of Infectious Disease, Faculty of Medicine, Imperial College London, London, UK; 3 Laboratorio de Investigación de Enfermedades Infecciosas, Laboratorios de Investigación y Desarrollo, Facultad de Ciencias y Filosofía, Universidad Peruana Cayetano Heredia, Lima, Perú; 4 Área de Investigación y Desarrollo, Asociación Benéfica PRISMA, Lima, Perú; 5 Department of International Health, Bloomberg School of Hygiene and Public Health, Johns Hopkins University, Baltimore, MD, USA; 6 Centro de Investigación en Enfermedades Tropicales “Maxime Kuczynski”, Instituto Nacional de Salud, Lima, Perú; 7 Instituto Peruano de Investigaciones en Salud, Loreto, Perú; 8 Dirección Regional de Salud, Loreto, Perú; 9 Department of Pediatric Cardiology, Children's Health Ireland at Crumlin, Dublin, Ireland; 10 St George's University Hospital NHS Foundation Trust, London, UK; 11 Nuffield Department of Primary Care Health Sciences, University of Oxford, Oxford, UK

## Abstract

Tuberculosis (TB) and soil-transmitted helminths (STHs) are major global health concerns, affecting approximately 1.5 billion and 1.8 billion people, respectively [1, 2]. Both are prevalent in poverty-stricken areas, and helminth infections are thought to polarise the immune response towards a type 2 response, possibly increasing susceptibility to TB [3, 4]. A major concern with TB is lung tissue destruction, which leads to high mortality and long-term morbidity [5]. In 2019, new active TB cases resulted in approximately 122 million disability-adjusted life years, with a projected economic cost of nearly USD 1 trillion from TB deaths between 2015 and 2030 [6, 7]. Individuals co-infected with TB and helminths may experience more severe lung damage compared to those with TB alone [8, 9].

## Shareable abstract

**Individuals with soil-transmitted helminth (STH) infection are more likely to have active pulmonary TB. Both TB and STH infections are independently associated with elevated plasma MMPs, which correlate with worse lung pathology.** https://bit.ly/3VgGWjm

*To the Editor*:

Tuberculosis (TB) and soil-transmitted helminths (STHs) are major global health concerns, affecting approximately 1.5 billion and 1.8 billion people, respectively [1, 2]. Both are prevalent in poverty-stricken areas, and helminth infections are thought to polarise the immune response towards a type 2 response, possibly increasing susceptibility to TB [3, 4]. A major concern with TB is lung tissue destruction, which leads to high mortality and long-term morbidity [5]. In 2019, new active TB cases resulted in approximately 122 million disability-adjusted life years, with a projected economic cost of nearly USD 1 trillion from TB deaths between 2015 and 2030 [6, 7]. Individuals co-infected with TB and helminths may experience more severe lung damage compared to those with TB alone [8, 9]. However, the underlying mechanisms are not well understood. Matrix metalloproteinases (MMPs), a family of zinc-containing enzymes, are biomarkers of lung extracellular matrix degradation. They are elevated in active TB and levels correlate with pulmonary destruction [10–12]. Helminth infections have also been shown to elevate MMPs, possibly due to tissue invasion and migration of these organisms [13]. We investigated the relationship between TB, STH infection, and these markers of lung tissue destruction. We examined the association between 1) TB, STH infection and circulating MMP concentrations and 2) circulating MMPs and the extent of TB lung damage.

This case–control study was conducted in Iquitos, a TB and STH endemic city in the Peruvian Amazon. We recruited adults aged 18 years and above, with smear or culture-confirmed pulmonary TB from 15 clinics in the city. Age- and sex-matched controls were recruited from the same neighbourhoods. Subjects were excluded if they had previous TB treatment, anti-helminth treatment within 6 months, or conditions potentially affecting immunity, including pregnancy, HIV, diabetes or cancer. People on immune-modulating medications, *e.g.* corticosteroids, were also excluded. All TB patients were tested for HIV. Participants completed a questionnaire covering socio-demographic characteristics and risk factors related to TB and STHs.

Blood samples were collected at enrolment, within 3 days of commencement of anti-TB therapy. Plasma MMP-9 levels and *Strongyloides* antibody were analysed by ELISA (R&D Systems, UK, and Bordier Affinity Products, Switzerland). MMP-1 and MMP-8 levels were analysed *via* magnetic microbead multiplex immunoassay (R&D Systems, USA). Three stool samples underwent copro-parasitology including direct microscopy, formol-ether concentration and modified acid-fast staining. TB patients’ sputum underwent microscopic-observation drug-susceptibility assay to confirm TB and check for drug resistance. If available, chest radiographs (CXRs) performed as part of routine TB care were graded by two independent reviewers blinded to helminth status using the TIMIKA score to document the extent of lung damage [14].

Participants were classified into: TB-positive, STH-positive (TB+STH+); TB-positive, STH-negative (TB+STH−); TB-negative, STH-positive (TB−STH+); and TB-negative, STH-negative (TB−STH−). Data were analysed using R Statistical software. Fisher's exact was used to test associations between categorical variables and Wilcoxon rank-sum for pairwise comparison of non-parametric data. Linear regression analyses were performed to determine association between STH positivity or STH polyparasitism and circulating MMP concentrations when adjusting for TB. Similarly, linear regression was performed to determine association between circulating MMPs or STH positivity and CXR severity score, controlling for age and smoking history. Ethics approval was obtained from the National Health Institute of Peru, participating hospitals, and Peru's Regional Health Directorate. Informed, written consent was obtained from all participants.

Among TB-positive participants, 40 of 61 (65.6%) had at least one STH infection compared to 22 of 51 (43.1%) TB-negative participants (p=0.02, Fisher's exact). *Strongyloides* was the most frequently detected STH followed by *Trichuris*, hookworm and *Ascaris* in both groups. There were no significant differences in age, sex, educational attainment, smoking/alcohol history, biomass fuel exposure or poverty index between TB-positive and TB-negative groups.

In pairwise comparisons (figure 1a), MMP concentrations were significantly elevated in STH-positive participants compared to STH-negative participants in all MMPs measured: MMP-1 (2717.11 *versus* 1407.87 pg·mL^−1^; p<0.01), MMP-8 (7824.66 *versus* 3712.90 pg·mL^−1^; p<0.01) and MMP-9 (329 000.00 *versus* 203 300.00 pg·mL^−1^ p<0.01). TB-positive participants typically had higher MMP concentrations compared to TB-negative participants, illustrated by red *versus* blue dots in figure 1a. Adjusting for TB-positivity in linear regression analysis, STH infection was independently associated with significantly higher concentrations of MMP-1 (beta 1.30, p=0.03) and MMP-9 (beta 1.50, p=0.02), and borderline significant for MMP-8 (beta 1.37, p=0.07). In the same model, now controlling for STH-positivity, TB was significantly associated with elevated concentrations of all MMPs measured (MMP-1 (beta 3.39, p<0.001); MMP-8 (beta 4.06, p<0.001); MMP-9 (beta 2.61, p<0.001). Beta coefficients represent the relative change in the outcome variable in the presence of the independent variable. For example, in STH infection, MMP-9 concentrations increase by a factor of 1.5, compared to baseline (STH-negative).

**FIGURE 1:**
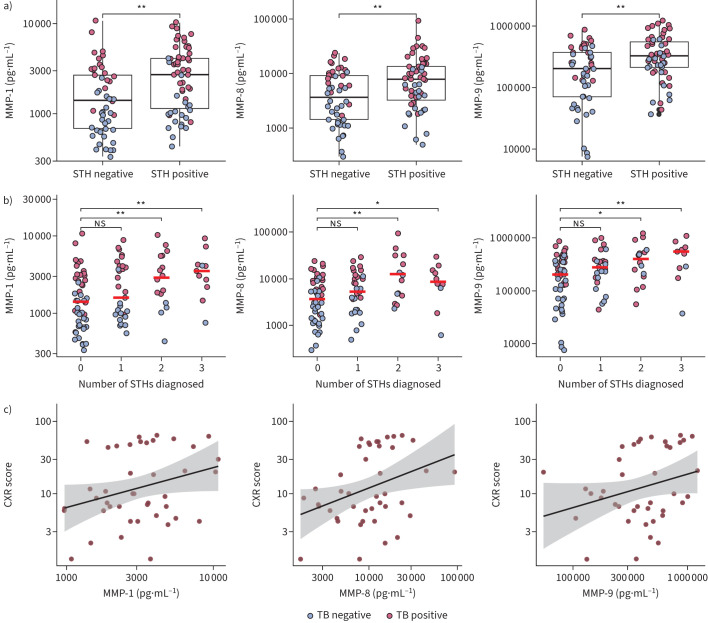
Relationship between matrix-metalloproteinases (MMPs), tuberculosis (TB) and soil-transmitted helminth (STH) positivity. a) Plasma MMP-1, -8 and -9 concentrations in STH-negative and -positive participants. Medians are shown with interquartile range (IQR; box), the whiskers represent 1.5×IQR (approximately 95% confidence interval). b) MMP-1, -8 and -9 concentrations in relation to number of STH species diagnosed. Scatterplot of MMP concentrations plotted against number of helminths diagnosed. Median is shown by a red bar. c) Correlation between chest radiograph (CXR) scores and MMP-1, -8 and -9 concentrations. Individual data points are seen in red; the standard error of the regression line is shown in shaded grey. For all plots, values are displayed in log_10_ scale for normalisation of non-parametric data and each circle represents a single individual: blue for TB-negative participants, red for TB-positive participants. Statistical significance was assessed by Wilcoxon rank-sum test. *: p<0.05; **: p<0.01; ns: nonsignificant.

MMP concentrations increased with number of STH species diagnosed (polyparasitism) (figure 1b) and linear regression modelling demonstrated a significant positive relationship for all MMPs when comparing individuals with 2+ STH species to those with no STH infection: MMP-1 (beta 1.35, p=0.04), MMP-8 (beta 1.61, p=0.03) and MMP-9 (beta 1.60, p=0.04).

Of the 40 TB-positive participants who had CXRs, 23 (57.5%) were STH-positive and 17 (42.5%) were STH-negative. The intra-class correlation coefficient (ICC(A,1)) was 0.99 (p<0.0001) indicating excellent agreement between independent reviewers. MMP concentrations positively correlated with CXR severity scores (figure 1c). Linear regression analysis confirmed a significant positive association between all measured MMPs and CXR scores: MMP-1 (beta 2.06, 95% CI 1.08–3.91, R^2^ 0.15), MMP-8 (beta 1.83, 95% CI 1.16–2.88, R^2^ 0.19) and MMP-9 (beta 1.82, 95% CI 1.00–3.31, R^2^ 0.12). STH status was not associated with CXR score in these linear models.

We found STH infection was associated with active pulmonary TB, with increased circulating MMPs seen in both TB and STH infection, and higher concentrations observed in individuals with more than one STH species diagnosed. While the influence of TB is clearly substantial, as previously observed by our group and others [10–12], our data support an independent association between STHs and MMPs and provide a plausible mechanism behind the observed increased tissue destruction seen in TB-STH co-infection. Reports on MMPs in STHs are scarce, although a murine model using the mouse hookworm *Nippostrongylus brasiliensis* showed up-regulation of MMP-12 associated with emphysematous changes [15]. Our findings are consistent with an Indian study of TB–*Strongyloides* co-infection which observed increased circulating MMP-1, -2, -7, -8 and -9 [10].

Increased MMP concentrations correlated with greater lung tissue destruction, as measured by CXR severity score and concurs with previous work done by our group and others [11, 12]. STH infection was positively and independently associated with plasma MMP concentrations, with increasing effect in polyparasitism, suggesting that for individuals with more STH species, there was a higher potential effect on lung damage. To our knowledge this is the first study examining the effect of polyparasitism on circulating markers of tissue destruction. Our data reinforces the concept that TB–STH co-infected individuals experience increased pulmonary damage, supported by studies from Brazil and India, which reported more advanced disease and increased risk of pulmonary cavitation in TB–*Strongyloides* co-infection [11, 13]. In addition, our findings shed new light on potential underlying mechanisms in tissue destruction in TB–STH co-infection.

Study limitations are that the cross-sectional design limits the ability to determine the temporal sequence of TB and STH infections. However, it is more plausible that STH infections occur first, facilitating TB development through immune modulation. Identifying active helminth infections is challenging, and positive *Strongyloides* serology might indicate past infections. The observed lack of association between STH status and CXR severity may be due to the limited CXR sample size. Finally, the study population was from a remote city in the Amazon, and the findings need validation in other settings.

This study found STH infection was associated with elevated plasma MMP concentrations, independent of TB status. This effect increased with polyparasitism, suggesting that individuals with more STH species had higher concentrations of circulating MMPs, markers of lung tissue destruction. Although increased MMPs are of uncertain clinical significance in STH mono-infection, they are likely to be pathogenic in TB–STH co-infection, or indeed any disease characterised by active pulmonary inflammation and ongoing matrix degradation. These findings indicate a possible mechanism underpinning the observation that TB–STH co-infected individuals appear to have more lung damage compared to those with TB alone. Our data highlight the importance of considering STH co-infection in TB endemic areas, with implications for screening and treatment. In particular, there are potential benefits from integrated TB and helminth control programmes.

## Shareable PDF

10.1183/13993003.01445-2024.Shareable1 This PDF extract can be shared freely online.
Shareable PDF ERJ-01445-2024.Shareable
