# Supplementary material for: Up-regulated matrix metalloproteinase activity in soil-transmitted helminth–tuberculosis co-infection is associated with increased lung pathology
Source: Eur Respir J. 2025 Jan 30;65(1):2401445. doi: 10.1183/13993003.01445-2024 (PMC11780722; doi:10.1183/13993003.01445-2024)
Supplement: Supplementary file 1 [file ERJ-01445-2024.Shareable.pdf]

SHAREABLE PDF

Up-regulated matrix metalloproteinase activity in soil-transmitted helminth–tuberculosis co-infection is associated with increased lung pathology

Maria-Cristina I. Loader [1,2,3], Sory Vasquez Alves [4], Robert H. Gilman [3,4,5], Jorge Coronel [3], Carmen Taquiri [3], Neusa Vasquez Alves [4], Fabiola Díaz-Soria [6], Salomón Durand [7,8], Sean T. Kelleher [9], Teresa Jacob [10], William H. Elson [11], Daniela E. Kirwan [1] and Jon S. Friedland [1]

[1]Institute for Infection and Immunity, St George's University of London, London, UK. [2]Department of Infectious Disease, Faculty of Medicine, Imperial College London, London, UK. [3]Laboratorio de Investigación de Enfermedades Infecciosas, Laboratorios de Investigación y Desarrollo, Facultad de Ciencias y Filosofía, Universidad Peruana Cayetano Heredia, Lima, Perú. [4]Área de Investigación y Desarrollo, Asociación Benéfica PRISMA, Lima, Perú. [5]Department of International Health, Bloomberg School of Hygiene and Public Health, Johns Hopkins University, Baltimore, MD, USA. [6]Centro de Investigación en Enfermedades Tropicales "Maxime Kuczynski", Instituto Nacional de Salud, Lima, Perú. [7]Instituto Peruano de Investigaciones en Salud, Loreto, Perú. [8]Dirección Regional de Salud, Loreto, Perú. [9]Department of Pediatric Cardiology, Children's Health Ireland at Crumlin, Dublin, Ireland. [10]St George's University Hospital NHS Foundation Trust, London, UK. [11]Nuffield Department of Primary Care Health Sciences, University of Oxford, Oxford, UK.

Corresponding author: Maria-Cristina I. Loader (mloader@sgul.ac.uk)

Check for updates

Shareable abstract (@ERSpublications)
Individuals with soil-transmitted helminth (STH) infection are more likely to have active pulmonary TB. Both TB and STH infections are independently associated with elevated plasma MMPs, which correlate with worse lung pathology. https://bit.ly/3VgGWjm

Cite this article as: Loader M-CI, Vasquez Alves S, Gilman RH, *et al*. Up-regulated matrix metalloproteinase activity in soil-transmitted helminth–tuberculosis co-infection is associated with increased lung pathology. *Eur Respir J* 2025; 65: 2401445 [DOI: 10.1183/13993003.01445-2024].

This PDF extract can be shared freely online.

Copyright ©The authors 2025.

This version is distributed under the terms of the Creative Commons Attribution Licence 4.0.

Received: 17 June 2024
Accepted: 27 Nov 2024

To the Editor:
